# Coupled Effects of Using Magnetic Field, Rotation and Wavy Porous Layer on the Forced Convection of Hybrid Nanoliquid Flow over 3D-Backward Facing Step

**DOI:** 10.3390/nano12142466

**Published:** 2022-07-18

**Authors:** Kaouther Ghachem, Fatih Selimefendigil, Badr M. Alshammari, Chemseddine Maatki, Lioua Kolsi

**Affiliations:** 1 Department of Industrial Engineering and Systems, College of Engineering, Princess Nourah bint Abdulrahman University, P.O. Box 84428, Riyadh 11671, Saudi Arabia; kgmaatki@pnu.edu.sa; 2 Department of Mechanical Engineering, Celal Bayar University, Manisa 45140, Turkey; fatih.selimefendigil@cbu.edu.tr; 3 Department of Electrical Engineering, College of Engineering, University of Ha’il, Ha’il City 81451, Saudi Arabia; bms.alshammari@uoh.edu.sa; 4 Department of Mechanical Engineering, College of Engineering, Imam Mohammad Ibn Saud Islamic University, Riyadh 11432, Saudi Arabia; casmaatki@imamu.edu.sa; 5 Department of Mechanical Engineering, College of Engineering, University of Ha’il, Ha’il City 81451, Saudi Arabia; 6 Laboratory of Metrology and Energy Systems, Energy Engineering Department, National Engineering School, University of Monastir, Monastir 5000, Tunisia

**Keywords:** flow separation, rotating surface, magnetic field, CFD, hybrid nanofluid, ANN

## Abstract

In the present study, the effects of using a corrugated porous layer on the forced convection of a hybrid nanofluid flow over a 3D backward facing step are analyzed under the coupled effects of magnetic field and surface rotation. The thermal analysis is conducted for different values of the Reynolds number (Re between 100 and 500), the rotational Reynolds number (Rew between 0 and 2000), the Hartmann number (Ha between 0 and 15), the permeability of the porous layer (the Darcy number, Da between $10^{-5}$ and $10^{-2}$) and the amplitude (ax between 0.01 ap and 0.7 ap) and wave number (N between 1 and 16) of the porous layer corrugation. When rotations are activated, the average Nusselt number (Nu) and pressure coefficient values rise, while the increment of the latter is less. The increment in the average Nu is higher for the case with a higher permeability of the layer. When the corrugation amplitude and wave number are increased, favorable impacts of the average Nu are observed, but at the same time pressure coefficients are increased. Successful thermal performance estimations are made by using a neural-based modeling approach with a four input-two output system.

## 1. Introduction

Flow separation characteristics are important to be considered in many engineering practices. Dynamics of drag forces, heat transfer (HT) and performance features are affected by the flow separation in various systems, and examples can be found in aerodynamics, turbo-machinery, building energy saving, power generation systems, cooling, thermal management and many more. Flow over a backward/forward facing step (BFS/FFS) includes separated flow regions, and the interaction between the main flow and established complex vortex patterns becomes complicated. The size of the vortex behind the step and separation dynamics are affected by many factors such as the Reynolds number (Re), step size, expansion ratio of the channel and other geometrical and operating parameters. In the experimental work of Armaly et al. [1], an LDA method was used for the analysis of the flow over a single BFS in a channel and reattachment lengths by using air for the Re range between 70 and 8000. They observed different flow regimes while numerical predictions were also conducted for the 2D BFS configuration. In the review work of Chen et al. [2], basic modeling features and parameters for the flow over BFS were discussed in depth, while different application examples were provided.They considered HT aspects and control design applications with future trends and challenges. Many studies considered the 2D flow over BFS with HT [3,4,5,6] and 3D aspects of BFS flow were studied in various sources [7,8,9,10,11].

Many different techniques including active, passive and hybrid ones have been proposed for the convective HT control considering separated flow over BFS. Flow pulsations [12,13,14], installing fins/objects [15,16,17], considering stationary rotating cylinders [18,19,20] and using nanofluids [21,22,23,24,25,26,27] are among some of the available methods. The nanofluids technology has been used in many energy technologies including solar power, refrigeration, power generation, cooling of electronics, energy storage and many others [23,28,29,30,31,32,33,34]. In the review work of Salman et al. [35], applications of hybrid nanofluids on the performance improvements of micro-scale BFS were explored by considering numerous experimental and numerical studies. The potential of using nanofluids in the convective HT enhancements for BFS flow were shown and future challenges were addressed. In another review study by Mohammed et al. [36], basic procedures for nanofluid preparation, its behavior, properties and application for convective HT of BFS flow were explored. Flow separation characteristics by using nanofluids were analyzed, while transport mechanisms and stability issues of the suspension were discussed in detail. Other works that considered the application of nanofluids in convective HT enhancement in BFS flow can be found in Refs. [37,38,39,40,41]. The rotational surface effects in convective HT control have been considered in many studies. The additional momentum and heat exchange by using speed controlled objects provide flow and HT control. A rotating cylinder has been used in several studies for convection control. The size, location and rotational speeds have been found as the most influencing parameters on the performance improvements [42,43,44]. The coupled effects of using nanofluid with rotational surface effects have been considered in various studies, while nanofluids have been reported to improve the thermal efficiency where the amount of enhancement depends upon the particle type and loading amount in the base fluid [45,46,47,48,49,50].

The application of a magnetic field (MaF) is relevant in many engineering practices such as in geothermal energy extraction, microfluidic pumps, coolers of nuclear reactors, metals processing, biomedical applications, convection and many more systems [51]. In the review study of Kabeel et al. [52], MaF applications in diverse engineering systems have been presented with the impacts on the HT and fluid flow. In applications where separated flow may be encountered such as flow over BFS, the MaF can suppress the flow recirculation and may improve the HT performance [53,54,55]. The MaF in convective HT applications has been used with nanofluids [56,57,58,59,60,61]. M’hamed et al. [62] performed an extensive review for the applications of MaF with nanofluids while two major challenges were addressed. The cost and stability issues were mentioned as the challenges. In another review study by Sheikholeslami and Rokni [63], convective HT of nanofluids under MaF effects were studied by using several numerical and analytical models. Single and two phase approaches of nanofluid were considered, while constant and variable MaF cases were analyzed. In separated flow with MaF, nanofluids were included in several studies, while further thermal efficiency enhancement were reported [64,65,66].

Porous inserts can also be utilized in convection control and HT performance improvements, while applications may be encountered in drying, chemical processes, filtering, thermal management and cooling [67]. Analytical methods have been used to analyze the porous layer–fluid systems with HT [68,69,70]. Combined utilization of magnetic field and porous layers for convective HT have been considered in several studies including mixed convection of lid-driven cavity [71], natural convective HT in an inclined cavity [72], transient natural convection with heat generation within a square enclosure [73] and many others [74,75,76]. Coupled effects of using nanofluids and MaF in porous media applications can be found in Refs. [77,78,79,80,81,82].

The present study focuses on analyzing the coupled effects of surface rotation, magnetic field and installation of a porous layer on the convective HT for flow over 3D BFS geometry. The porous layer is a wavy one installed near the abrupt area change, while it has a rectangular type corrugation. Flow separation dynamics and convective HT performance are analyzed under coupled MaF effects, surface rotation and hybrid nanofluids. The mentioned methods may be already available in the system such as rotating heat exchanger with area expansion, or MaF effects may also be present. In the literature, convective HT control by using the combined effects of MaF, rotations and installation of a wavy porous layer has never been considered for flow in a channel with area expansion. A neural modeling approach is used for fast thermal predictions of HT and pressure coefficients with different influencing input parameters for assisting the high fidelity computational fluid dynamics (CFD) simulations. As thermal management and flow control for channels with area change are important to be considered in many thermal engineering systems such as heat exchangers, electronic cooling, micro-fluidic devices and many more, the outcomes of the present work will be helpful for optimization and energy efficient product development in various energy system technologies.

## 2. Computational Model

### 2.1. Thermo-Fluid System and Governing Equations

**Figure 1:**
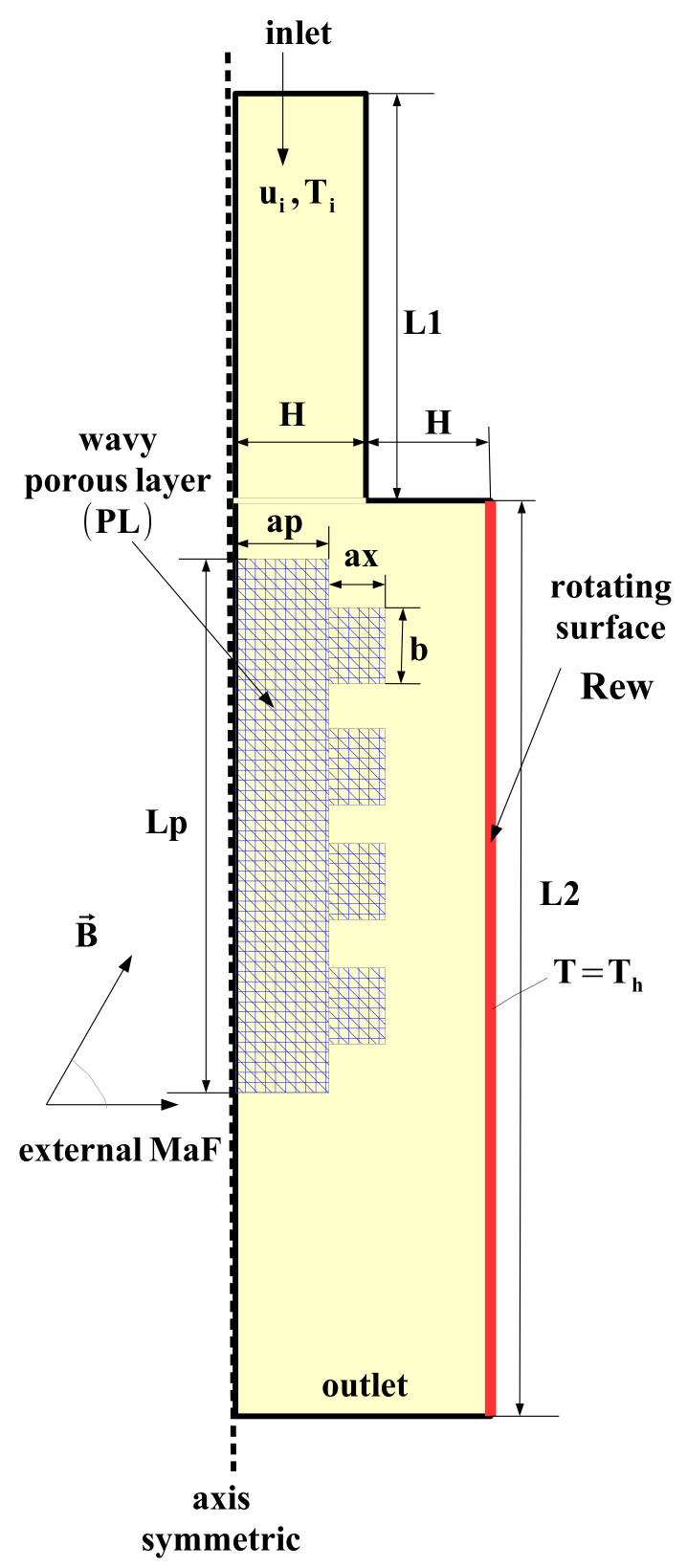
Schematic view of flow over BFS with wavy PL under coupled effects of rotations and MaF.

The effects of using a wavy PL and surface rotation on the flow separation and HT characteristics for hybrid nanofluid flow over 3D BFS is analyzed under MaF effects. Figure 1 shows the schematic view of the thermo-fluid system with the installed PL. The inlet distance to the step is L1, while L2 and H denote the distance of step to exit and step height. The inlet channel height is H. A wavy PL is used with length of Lp, and its thickness is ap. The form of the corrugation is a rectangular type with height of ax and length b, where N is the number of corrugation waves. The cold fluid stream enters with velocity of ui and cold temperature of Ti. The wall downstream of the step is at hot temperature of Th while it is also rotating with speed of ω. A water–Ag–MgO hybrid nanofluid is selected as the HT fluid. The correlations for the viscosity and thermal conductivity of the hybrşd nanofluid can be found in Ref. [83]. We considered a single phase approach of the nanofluid modeling, while fluid is Newtonian. The nanoparticle loading amount in the base fluid fluid is 2%. The MaF is acting radially, and it is uniform throughout the computational domain.

Forced convection of hybrid nanofluid for flow over 3D BFS is considered, while effects of thermal radiation, free convection and viscous dissipation are neglected. The induced MaF effects, joule heating and displacement currents effects are also ignored. In the PL domain, the Brinkman-extended Darcy porous model is considered. The governing equations in compact form are stated as in the following [84,85]:

(1) $$\nabla .\vec{u}=0$$

(2) $$\frac{1}{\varepsilon^{2}}\left(\vec{u}.\nabla \right)\vec{u}=-\frac{1}{\rho }\nabla p+\frac{\nu }{\varepsilon }\nabla^{2}\vec{u}-\frac{\nu }{\kappa }\vec{u}+\frac{\sigma }{\rho }\left(\vec{u}\times \vec{B}\right)\times \vec{B}$$

(3) $$\left(\vec{u}.\nabla \right)T=\alpha \nabla^{2}T.$$

In the above given equations, Lorentz forces due to the imposed MaF are obtained. A uniform MaF acting only in the radial direction is considered with contributions as [86]:

(4) $$F_{m,r}=0,\;\;F_{m,z}=-\sigma wB_{r}^{2}.$$

Non-dimensional parameters are the Re, Rew, Da and Ha which are described as in the following:

(5) Re=uiHρμ,Rew=ω(H)2ρμ,Da=κH2,Ha=BHσρν.

The solid volume fraction of particles in the hybrid nanofluid is taken as 0.02. The temperature and velocity at the inlet are uniform with the values of $T_{i}$ and $u_{i}$. An axis–symmetrical model is used with $\frac{\partial T}{\partial r}=0$. Pressure outlet boundary condition is used at the channel exit. The bottom wall downstream of the step is maintained at a hot temperature of $T_{h}$. Other walls are considered to be adiabatic. The hot surface is also rotating with a speed of ω. Here, the tangentially moving wall velocity is 0 and the angular component is ωr. At the interface between the fluid and PL, the heat flux continuity is considered as: ${\left(k\frac{\partial T}{\partial n}\right)}_{p}={\left(k\frac{\partial T}{\partial n}\right)}_{f}$.

### 2.2. Solution Method

The GWR-finite element method (FEM) is selected as the solution method. The foundations and theory of FEM for fluid flow and HT problems can be found in several reference textbooks [87,88,89]. Successful applications of FEM in convective HT with separated flow have been reported [90,91,92].

In the method, field variables (*g*) approximation is made by using with different ordered Lagrange FEM as:

(6) $$g=\sum_{r=1}^{N^{s}}\Psi_{r}^{s}G_{r}$$

where $\Psi^{s}$ and $G_{r}$ denote the shape function and nodal value. The weighted average of the residuals is set to be zero as:

(7) $$\int_{V}WRdV=0$$

with *W* denoting the weight function and *R* as the residual. To handle local numerical instabilities, artificial diffusion with the streamline upwind Petrov–Galerkin method (SUPG) is considered, while the Biconjugate gradient stabilized iterative method solver (BICGStab) is utilized for fluid flow and heat transfer modules of the solver. Convergence criteria of 10${}^{-7}$ is used.

### 2.3. Grid Independence and Code Validation

**Figure 2:**
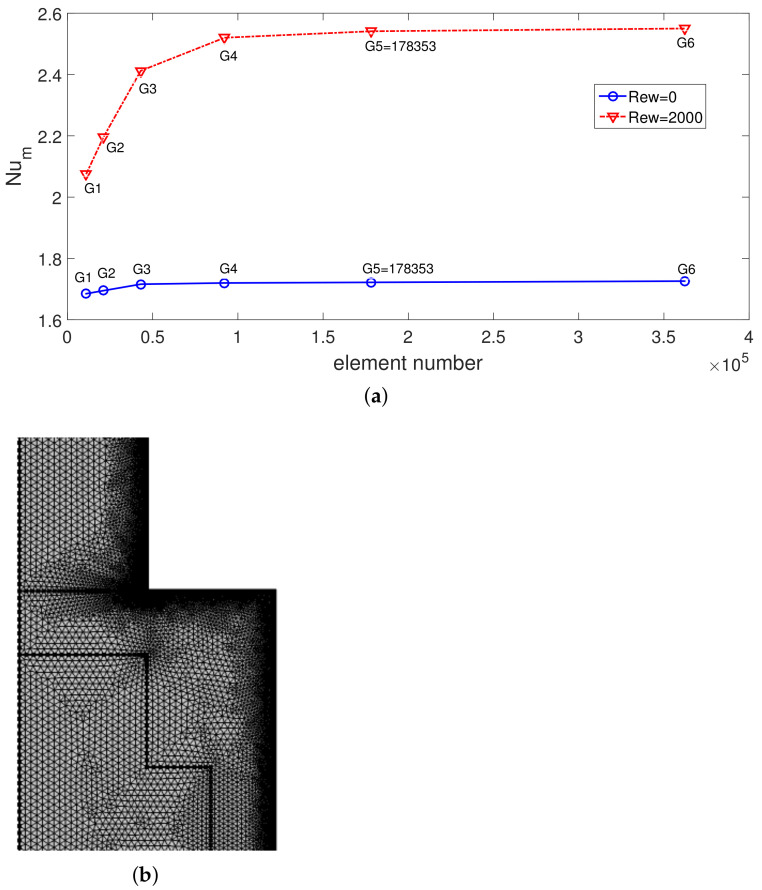
Numerical test results for grid independence at two Rew (**a**) (Re = 250, Da = 10${}^{-1}$, Ha = 7.5, ax = 0.25 ap, N = 4) and distribution of grid (**b**).

Grid independence of the solution is assured, and test results are given in Figure 2a. The average Nu for different grid sizes at Rew = 0 and Rew = 2000 are shown in the plot. A grid system G5 with 178,353 number of elements is used. Figure 2b shows the grid distribution near the step, while refinement is performed at the interfaces and towards the walls.

**Figure 3:**
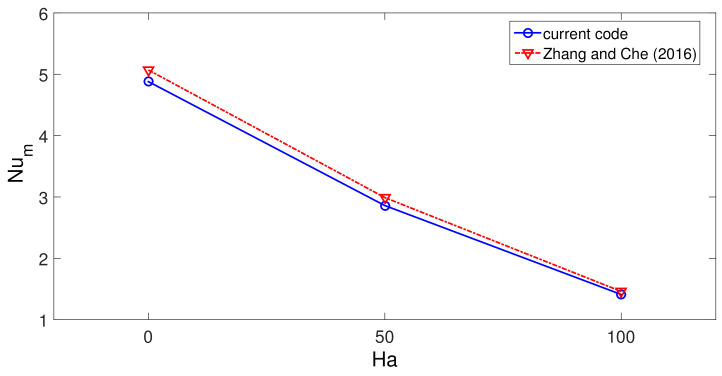
Code validation 1: Average Nu comparisons of convection in a differentially heated cavity for different Ha at Grashof number of $2\times 10^{5}$.

**Figure 4:**
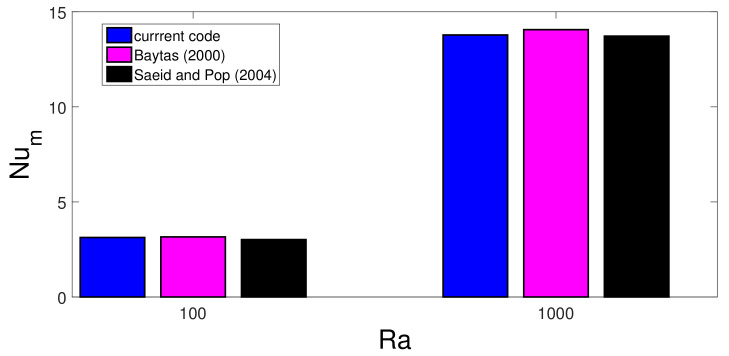
Code validation 2: Average Nu comparisons available in different sources for convective HT in a differentially heated porous cavity at two different Rayleigh numbers.

**Figure 5:**
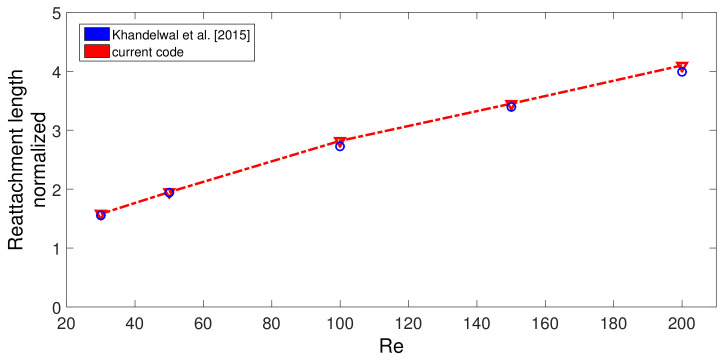
Code validation 3: Comparisons of normalized reattachment length for different Re for the flow in a T-branching channel by using the results in Ref. [96].

The code is validated by various sources available in the literature. In the first validation study, the effects of using MaF on convective HT in a differentially heated cavity is considered, and results in [93] were utilized. Comparisons of average Nu for three different MaF strengths at Grashof number of $2\times 10^{5}$ are shown in Figure 3. The highest difference below 5% is achieved between the results. In the second validation study, convection in a differentially heated porous enclosure is considered. Average Nu at two different Rayleigh numbers are shown in Figure 4 with the available results in two different sources [94,95]. As the last validation study, flow separation in a bifurcating T-channel is analyzed under laminar flow conditions. Figure 5 shows the comparison results of normalized reattachment length versus Re available in Ref. [96] for a Newtonian fluid. These results show that the present solver is capable of simulating the effects of MaF and porous media impacts on convective HT, while predictions for flow separation effects can also be performed.

## 3. **Results and Discussion**

The flow separation effects for a nanofluid over a BFS under the combined effects of using a wavy porous layer, MaF and surface rotation are numerically assessed. A rectangular type of corrugation is considered for the PL, while the part downstream of the step is considered to be hot and rotating. The thermo-fluid configuration is analyzed for different values of the Reynolds number (Re: 100–500), the rotational Reynolds number (Rew: 0–2000), MaF strength (Ha: 0–15), permeability of the medium (Da: $10^{-5}–10^{-2}$) and amplitude (ax: 0.01 ap: 0.7 ap) and eave number (N: 1–16) of the PL corrugation. Performance estimations are conducted by using NNets.

**Figure 6:**
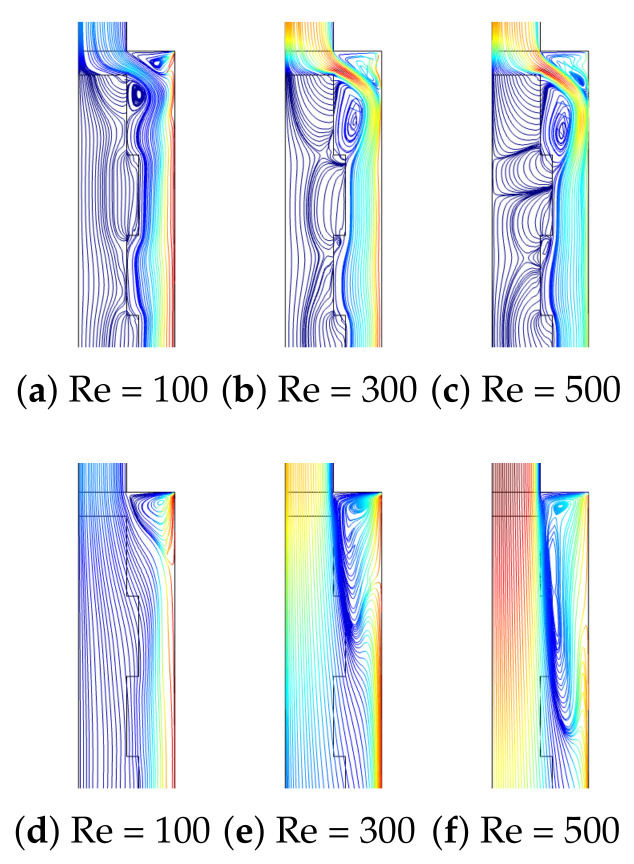
F-P variations with varying Re at two different permeabilities of the PL ((**a**–**c**), Da = 10${}^{-5}$), ((**d**–**f**), Da = 10${}^{-1}$) (Rew = 1000, Ha = 7.5, ax = 0.25 ap, N = 4).

**Figure 7:**
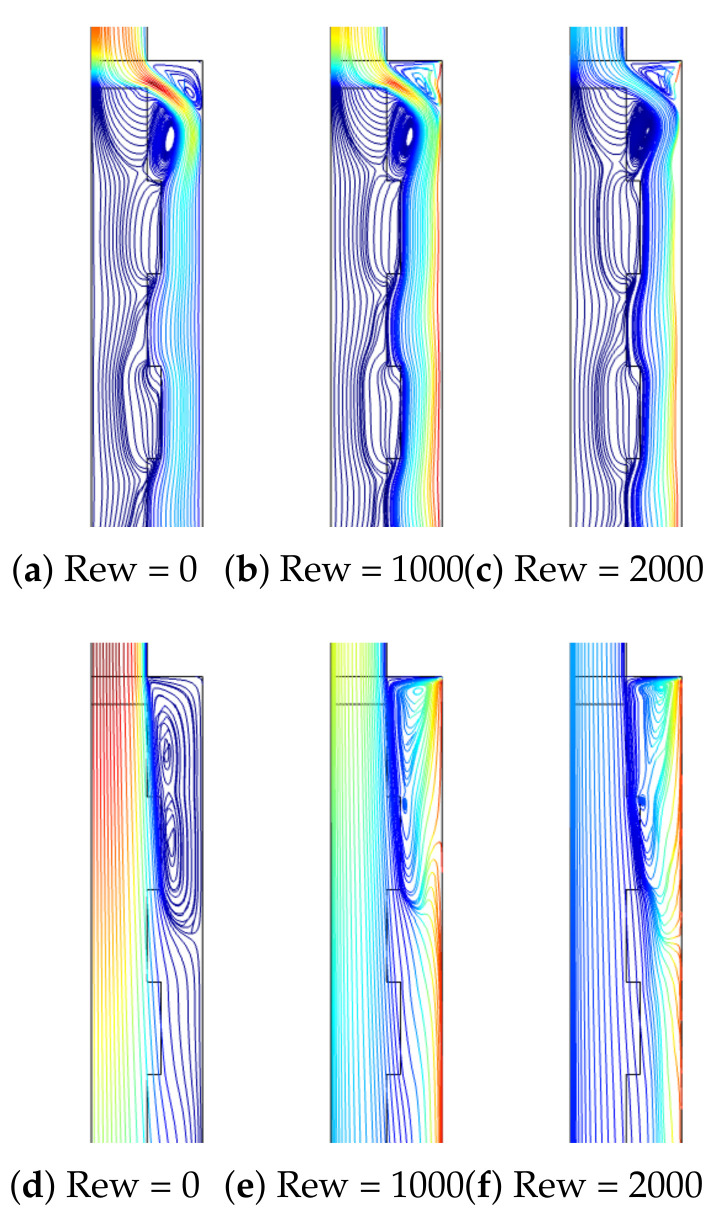
Effectsof Rew on F-P variations at two different permeabilities of the PL ((**a**–**c**), Da = 10${}^{-5}$), ((**d**–**f**), Da = 10${}^{-1}$) (Re = 250, Ha = 7.5, ax = 0.25 ap, N = 4).

Distributions of flow patterns for different Re are given in Figure 6 at two different permeability values of the PL for fixed values of (Rew = 1000, Ha = 7.5, ax = 0.25 ap, N = 4). For the lowest value of permeability, the PL resists more to flow, while the presence of corrugation becomes effective. The main fluid stream flows below the wavy layer, while a recirculation zone is established behind the step and another one above the main stream. As the value of Re is increased, the size of the vortices increases. However, the increment in the vortex size behind the step is significant with higher Re when the PL with a higher permeability is considered since in this case, the resistance of the PL to the main flow stream is minimum. The reattachment size extends from 0.5 H to Hat the lowest permeability while it extends from 1.5 H to 5.1 H at the highest permeability when Re is increased from Re = 100 to Re = 500. The presence of the surface rotation of the hot wall downstream of the step affects the vortex distribution behind the step which is significant when the PL with higher permeability is considered. The reattachment length size reduces from 3 H to 1.7 H when the rotations are activated from Rew = 0 to Rew = 2000. Near the walls behind the step, fluid velocity rises due to the rotation, while the main fluid stream is affected, and the vortex size is reduced (Figure 7).

**Figure 8:**
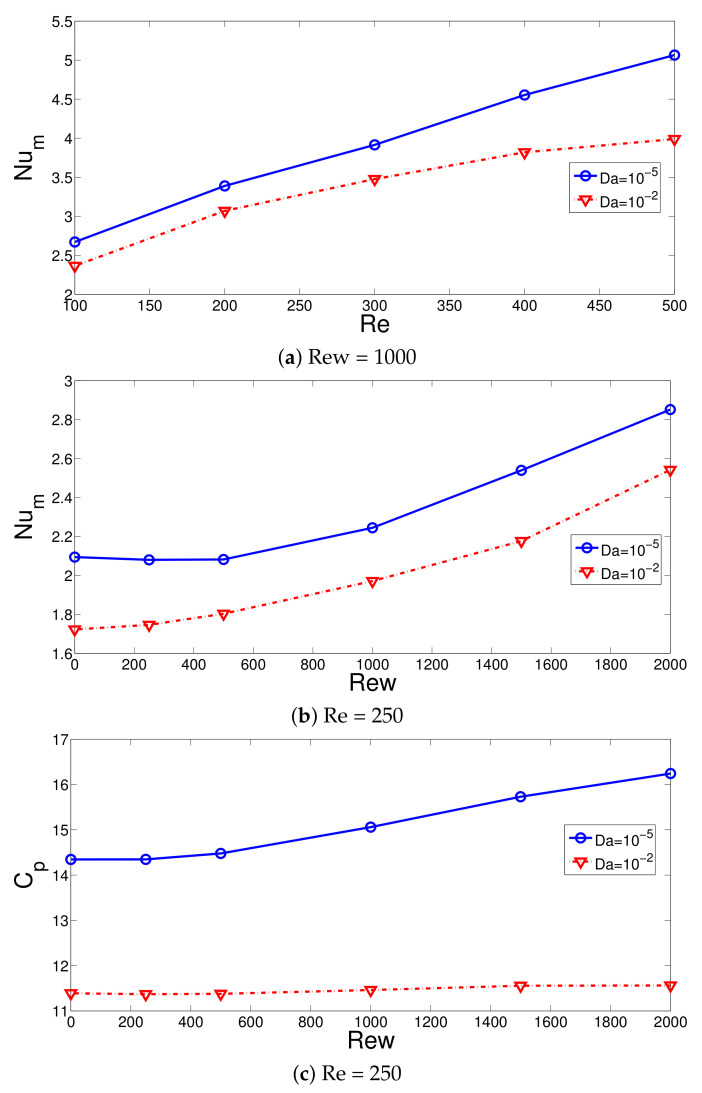
Effects of Re on the average Nu variations at two different permeabilities of the PL (**a**) and effects of Rew on the variation of average Nu (**b**) and pressure coefficient (**c**) (Ha = 7.5, ax = 0.25 ap, N = 4).

The average Nu rises with higher values of Re and Rew considering the lowest and highest PL permeability as shown in Figure 8a,b with (Ha = 7.5, ax = 0.25 ap, N = 4). The Nu values are higher when PL with lower permeability is considered. This is attributed to the more deflection of the main fluid stream toward the hot wall for the object with the lower permeability. At Re = 100, the Nu values are 13% higher when cases with the lowest and highest Da are compared, while this amount is further increased to 25% at Re = 500. The additional momentum added to the main stream and suppression of the recirculation with higher rotational speeds resulted in higher average HT values. The rise in the average Nu becomes 47.6% and 42.5% when the cases at Rew = 0 and Rew = 2000 are compared for the PL with the highest and lowest permeability. The vortex suppression with rotation is significant for the case when PL with lower permeability is considered. The pressure coefficient also rises with higher rotational speeds of the surface when PL with lower permeability is considered (Figure 8c). In this case, the value of Cp rises by about 13%, while less than 2% variation is seen when configuration at Da = $10^{-2}$ is considered.

**Figure 9:**
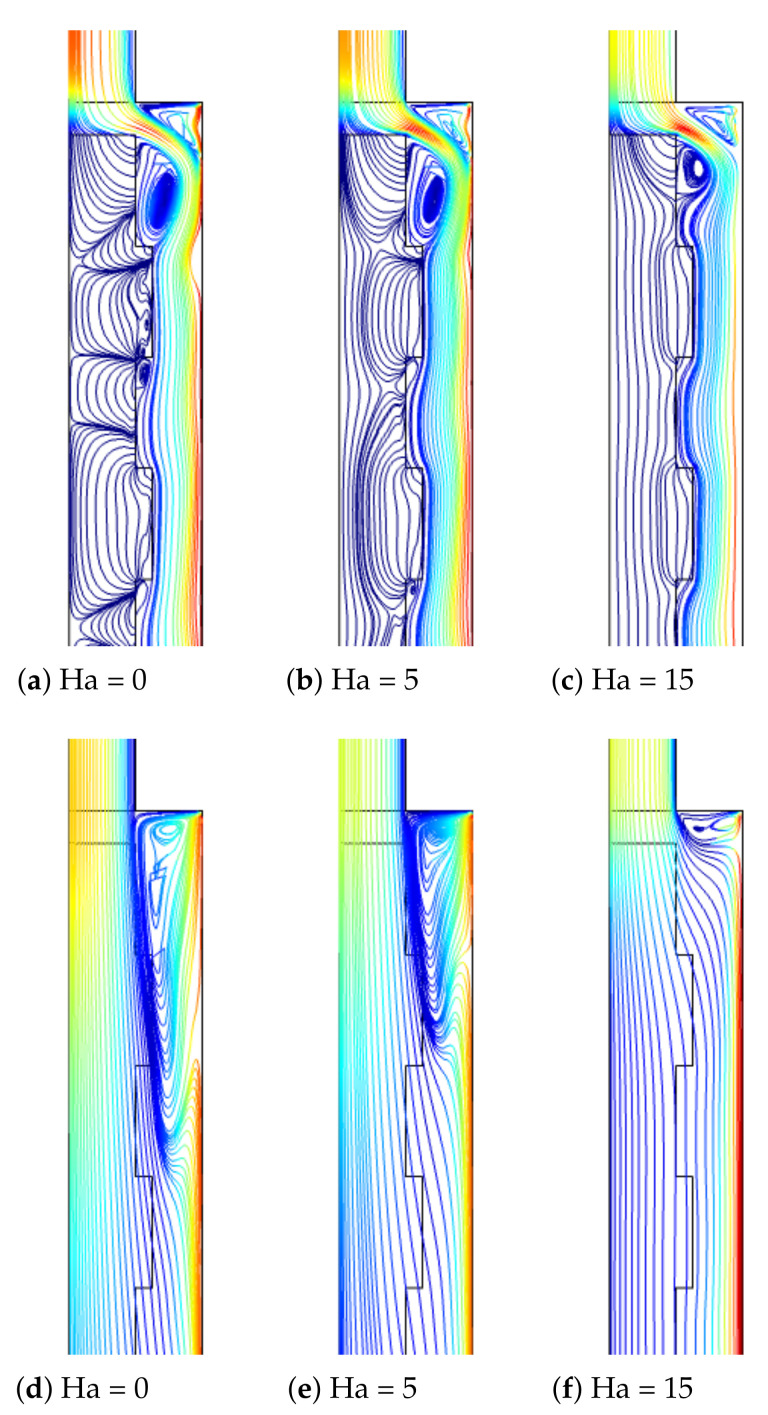
Impacts of MaF strength on the FP distributions at two different PL permeability ((**a**–**c**), Da = 10${}^{-5}$), ((**d**–**f**), Da = 10${}^{-2}$) (Re = 250, Rew = 1000, ax = 0.25 ap, N = 4).

**Figure 10:**
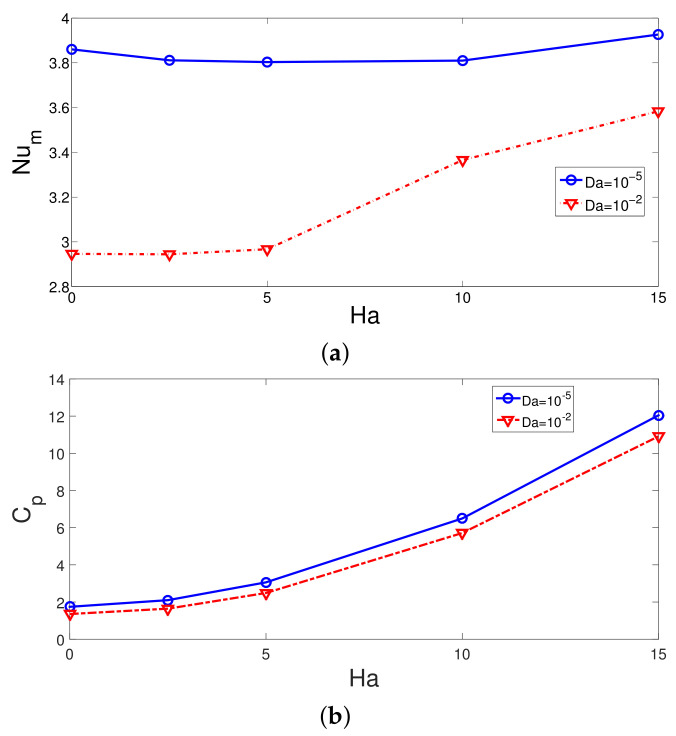
Average Nu and pressure coefficient variations for different MaF strengths at two different PL permeabilities (Re = 250, Rew = 1000, ax = 0.25 ap, N = 4).

MaF has the potential to suppress large recirculation regions and to enhance the HT coefficient which has been shown in previous studies of convective HT. In this study, an external MaF in radial radiation is imposed which is uniform throughout the computational domain. The impacts of Ha on the FP variations are shown in Figure 9 for the flow over 3D BFS having the PL at two different permeabilities with fixed values of (Re = 250, Rew = 1000, ax = 0.25ap, N = 4). The vortex size reduction is very effective for the case when PL with the highest permeability is considered, while a large recirculation zone is established behind the step for this configuration. However, for the case with PL at Da = $10^{-5}$, the vortex size behind the step is slightly affected with MaF strength. The average Nu rises with higher MaF strength at Da = $10^{-2}$, while at the highest Ha, the amount of increment is 21.8%. However, for the configuration with PL permeability of Da = $10^{-5}$, the variation in the average Nu is less than 2% when varying the MaF strength. The pressure coefficient is higher for the case with lower permeability of PL. The discrepancy between the Cp values becomes 28% in the absence of MaF and 11% at Ha = 15 when cases of lowest and highest permeability are compared (Figure 10).

**Figure 11:**
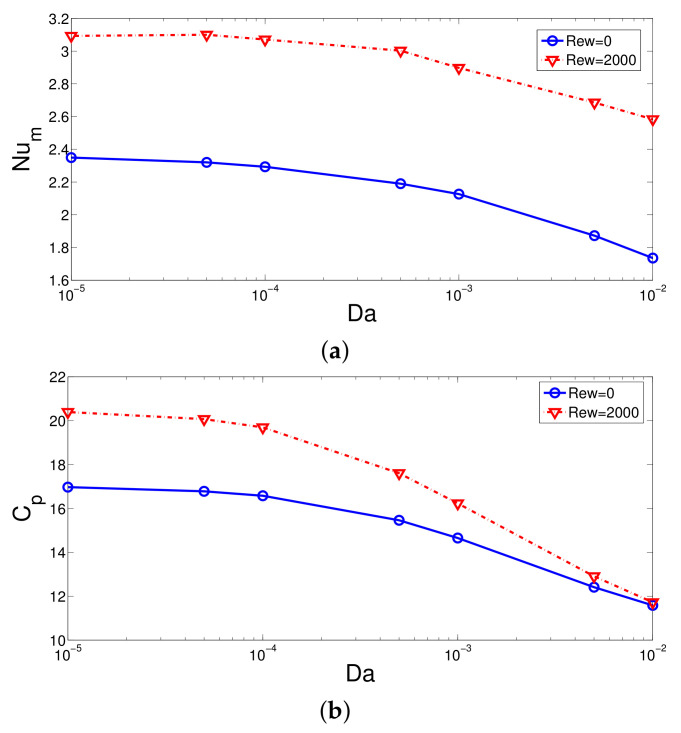
Effects of PL permeability on the variation of average Nu (**a**) and pressure coefficient (**b**) for stationary and rotating surface (Re = 250, Ha = 7.5, ax = 0.25ap, N = 4).

Comparisons of average Nu and pressure coefficients for different Da at stationary and rotating wall cases are shown in Figure 11 for fixed values of (Re = 250, Ha = 7.5, ax = 0.25 ap, N = 4). Lower permeability configurations with rotating surfaces lead to higher HT and pressure coefficients. The impacts of rotation of the variation of Cp reduces with permeability. The impacts of rotation on the reduction of average Nu with higher permeability becomes less. The amount of reduction in the average Nu between the lowest and highest Da cases becomes 14% and 52.7% at Rew = 2000 and Rew = 0.

**Figure 12:**
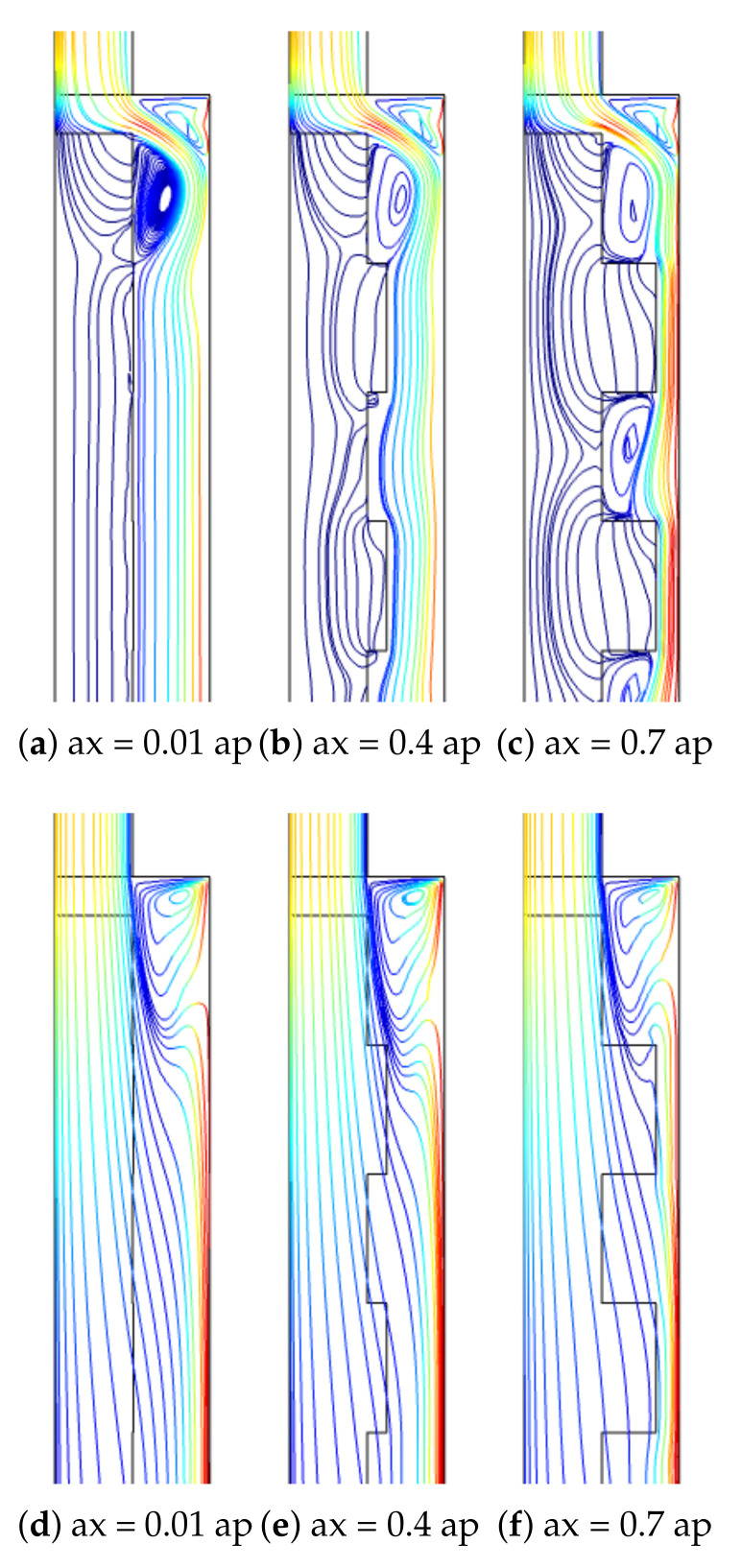
Effects of corrugation height of the PL at two different permeabilities ((**a**–**c**), Da = 10${}^{-5}$), ((**d**–**f**), Da = 10${}^{-2}$) (Re = 250, Rew = 1000, Ha = 7.5, N = 4).

The PL is a wavy one with rectangular type corrugation. The corrugation height is denoted by ax, while the number of corrugation waves is denoted by N. Corrugation wave amplitude effects on the flow pattern variations are shown in Figure 12 for two permeabilities of the PL at (Re = 250, Rew = 1000, Ha = 7.5, N = 4). The impact of corrugation height is effective for the case with lower permeability, while vortices are formed in the small cavities of the corrugation at the interface between the PL and fluid.

**Figure 13:**
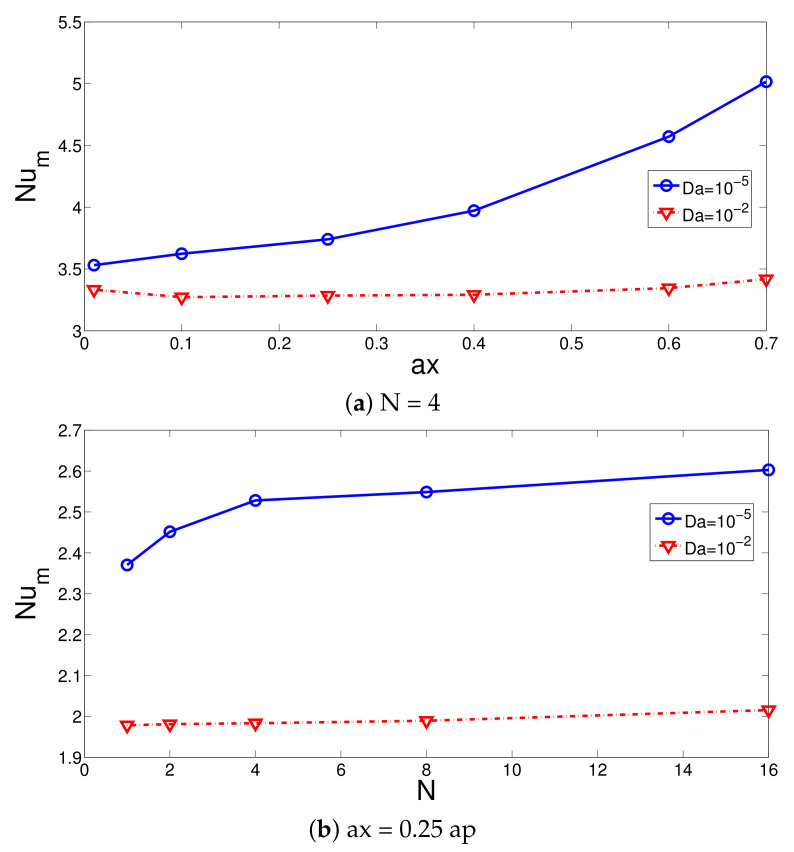
Corrugation amplitude (**a**) and wave number (**b**) on the average Nu variations (Re = 250, Rew = 1000, Ha = 7.5).

**Figure 14:**
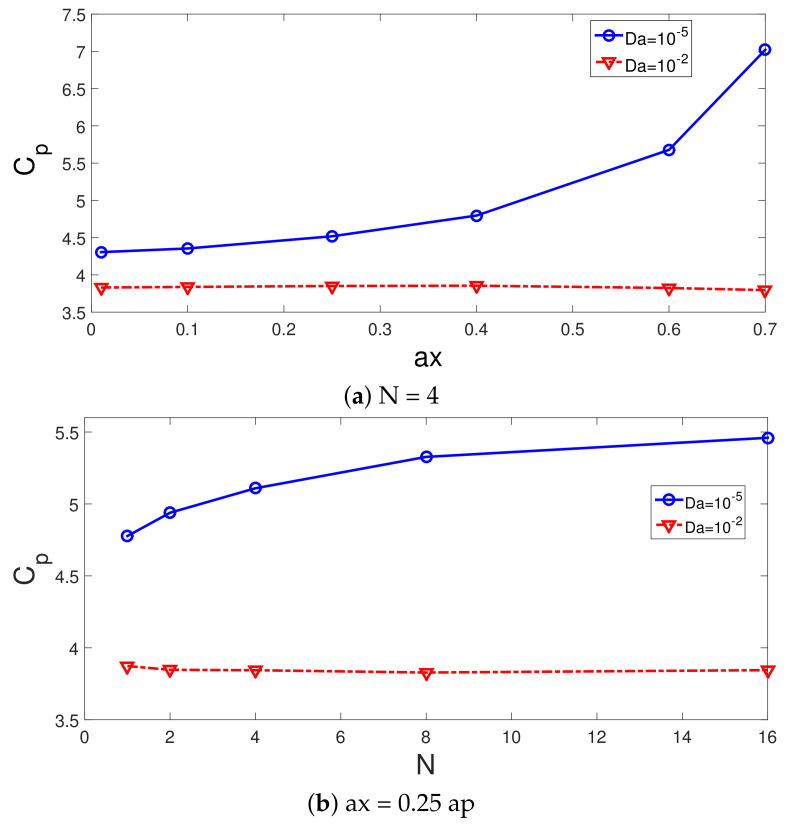
Pressure coefficient variations with different corrugation amplitude (**a**) and wave number (**b**) at two different PL permeabilities (Re = 250, Rew = 1000, Ha = 7.5).

The impacts of the corrugation geometric parameters on the average Nu and pressure coefficients are effective for the case with the lower permeability of the PL as shown in Figure 13 and Figure 14 with (Re = 250, Rew = 1000, Ha = 7.5). A higher value of the corrugation height results in higher HT and pressure coefficients. This is due to the reduction in the gap between the rotating hot surface and corrugated PL interface where at lower values of Da, local fluid velocity rises, and thermal transport enhances. The PL acts as an obstacle that redirects the main fluid stream toward the hot wall for lower permeability. The HT and pressure coefficients rise by about 42% and 62.8% when increasing the wave amplitude from ax = 0.01 ap to ax = 0.7 ap. However, the amount of rise becomes only 9.7% and 14.46% when the wave number is increased from N = 1 to N = 16. It is seen that the hydro thermal performance can be controlled in a good way by varying the permeability and wavy geometrical parameters of the PL. The permeability of the PL and corrugation amplitude have the highest impacts of the HT and pressure coefficient variations.

Artificial neural networks (ANNs) and other soft computing methods are commonly used in thermal engineering systems for fast performance predictions and in assisting the high fidelity parametric computational fluid dynamics (CFD) simulations. Correlations for the HT and pressure coefficients can be devolved based on neural modeling. In the present study, the trends in the curves of average Nu and pressure coefficient (Cp) for varying Re, Rew, Da and Ha are captured by using a mathematical model based on NNs. The range of parameters are taken as: (Re between 100–500), (Rew between 0–2000), (Ha between 0–15), (Da between $10^{-5}$–$10^{-2}$). Table 1 shows the input variable names and their ranges. The basic steps and procedures of the NN based modeling can be found in different textbooks [97,98,99]. Output is given for the neuron modeling of NNs:

(8) $$u_{j}=\sum_{i=1}^{N}w_{ij}y_{i}$$

where inputs and weights are denoted by $y_{i}$ and $w_{ij}$. The model structure of NN has different layers; input layer, output layer and at least one hidden layer. Each layer weights are used for the connections, while each unit sums its inputs and bias terms. Mean squared error (MSE) and coefficient of determination ($R^{2}$) are commonly used as the NN performance indices which are given as:

(9) $$\mathrm{MSE}=\frac{1}{N}\sum_{i=1}^{N}{\left(y_{i}-y_{i}^{*}\right)}^{2}.$$

(10) $$R^{2}=1-\frac{\sum_{i=1}^{M}{\left(y_{i}-y^{*}\right)}^{2}}{\sum_{i=1}^{M}{\left(y_{i}-{\bar{y}}^{*}\right)}^{2}}.$$

The data set number is given by *N*, while the average value is represented by $\bar{y}$.

**Figure 15:**
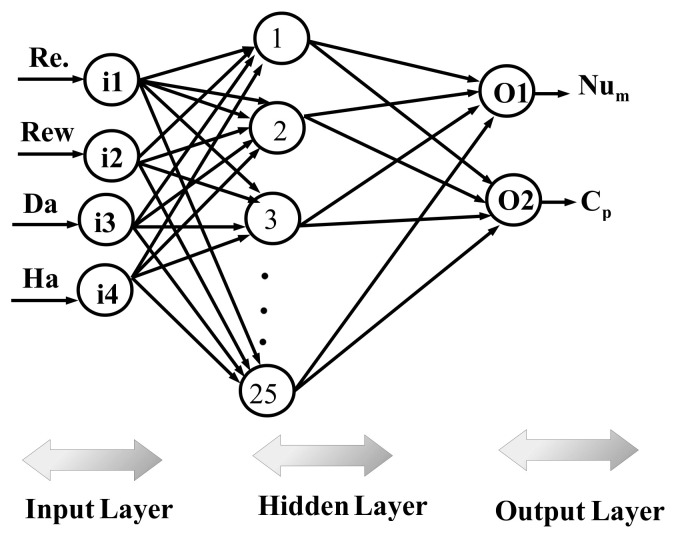
A schematic view of the NN structure with inputs, outputs and layers.

Updating of the weights is accomplished during the training of the NN, while the MSE or other performance metrics are utilized. The schematic view of the NN with the input–output data set and different layers is given in Figure 15.

**Figure 16:**
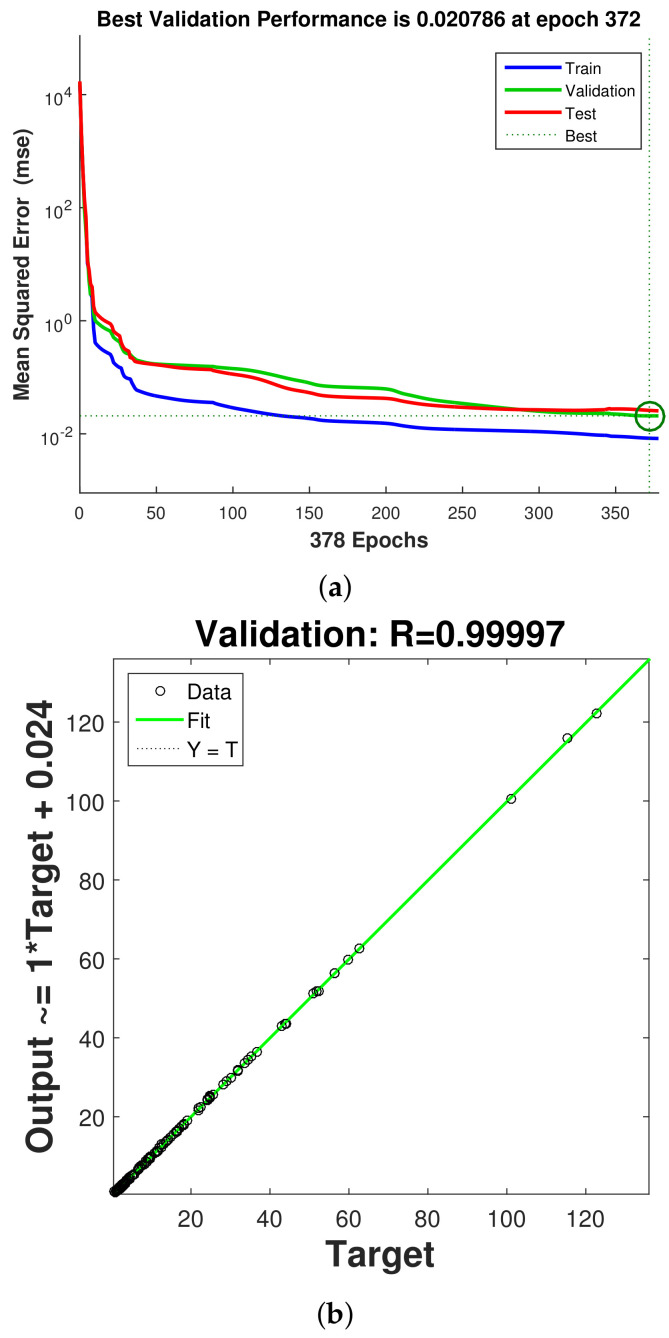
NN performance with different iterations for different data sets (**a**) and regression plot for validation data set (**b**).

**Figure 17:**
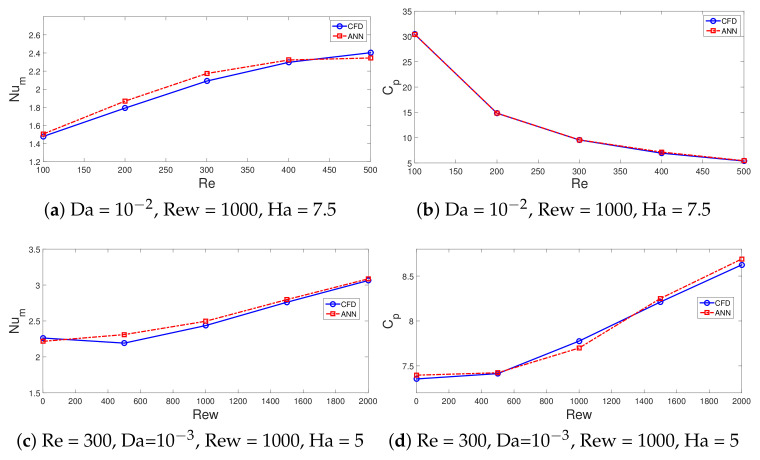
Comparison of average Nu (**a**,**c**) and pressure coefficients (**b**,**d**) variations obtained with CFD and NN.

**Figure 18:**
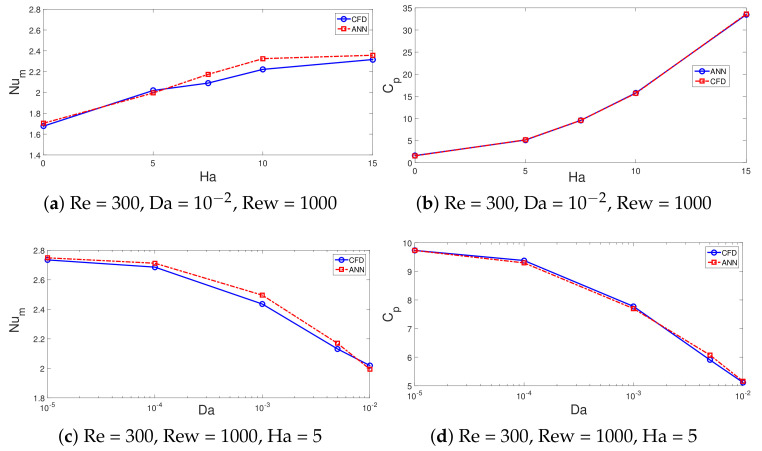
Comparisons of average Nu and pressure coefficient variation with MaF strength (**a**,**b**) and permeability of PL (**c**,**d**) obtained with CFD and NNs.

In total, a 2401 data set from CFD simulations is used, while data is divided randomly into training, validation and testing data sets; 70% of the data is used for training, while the remaining 15% of each data set is used for validation and testing. One hidden layer is used with the hyperbolic tansig as the activation function ($f\left(x\right)=1/\left(1+e^{\left(-x\right)}\right)$). The hidden layer has 25 neurons, while Levenberg–Marquardt with backpropogation is considered as the learning algorithm of NN [100,101]. An initial value of 10 neurons in the hidden layer is considered, and performance of the NN is explored by using different numbers of neurons, while NN structure with 25 neurons gives the best performance. Table 2 shows the neural model properties. The performance of NN is shown in Figure 16a. During the initial stage of training, the MSE value is high, while after the iteration proceeds, its value is further reduced and best performance is achieved at epoch 372. Figure 16b presents the regression plot for the validation data set. The correlation coefficient is high, while the scattering of points is minimum. Table 3 presents the performance of the neural network model for different data sets. The variations of the average Nu and pressure coefficients with change in the parameters of interest (Re, Rew, Ha and Da) estimated by the NN approach are given in Figure 17 and Figure 18. The trends in the curves of average Nu and Cp are very well captured with the NN procedure. The model has a mathematical form in terms of input–output relations (four input–two out system) via network weights and bias terms which allow fast estimation results for the parameters of interest within the range.

## 4. Conclusions

Effects of surface rotation, MaF and installation of a wavy porous layer on the convective HT of hybrid nanofluid flow in a 3D channel with area expansion are examined. The following conclusions can be drawn:

- The average Nu increases as the value of Re and Rew increase, while the HT values are higher for the case when the PL with lower permeability is installed. The Nu values are 13% higher at Re = 100, while they are 25% higher at Re = 500 when the cases between the lowest and highest PL permeability are compared.
- The average Nu increment up to 47.6% is obtained when the rotation of the surface is activated at Rew = 2000. The suppression of the vortex behind the step with rotation is profound for the PL with higher permeability, while the pressure coefficient rises by about 13%
- When MaF is imposed, significant impact on the vortex suppression is seen for the case with higher permeability of the PL. The average Nu rises by about 21.8% at the highest MaF strength at Da = $10^{-2}$, while it is less than 2% at Da = $10^{-5}$.
- In the absence and presence of MaF effects, by using the PL with the lowest and highest permeability, the difference between the pressure coefficient becomes 28% and 11%.
- By using wavy PL and varying its parameters, convective HT control can be achieved while highest effects on the thermal performance improvements are achieved.
- Wave amplitude is more influential on the thermal performance improvement as compared to wave number of the corrugation. When wave corrugation amplitude rises, the increments in the average Nu and Cp become 42% and 62.8%. However, when the wave number is increased from 1 to 16, they increase by about 9.7% and 14.46%.
- Thermal performance estimations are made by using 25 neurons in the hidden layer. The ANN model with four inputs (Re, Ew, Da, Ha) and two outputs (average Nu, pressure coefficient) is considered. Fast and accurate thermal predictions are made with neural modeling of convective flow over 3D BFS considering the coupled effects of MaF, surface rotation and installation of a wavy porous layer.

The present work can be extended to include different wave forms, non-uniform MaF effects, and channel expansion ratio effects. Different soft computing techniques such as ANFIS (Adaptive-Network Based Fuzzy Inference Systems), SVM (Support Vector Machines) can also be used and their performances can be compared with ANN model performance.

## Figures and Tables

**Table 1:** Input–output data set.

Parameter Name	Range	Number of Values
Reynolds number (Re)	100–500	7
rotational Reynolds number (Rew)	0–2000	7
permeability of porous layer (Da)	$10^{-5}$–$5\times 10^{-2}$	7
magnetic field strength (Ha)	0–15	7

**Table 2:** NN model properties.

Property	Name-Value
data division	dandom
training algorithm	Levenberg-Marquardt
performance metric	mean squared error (MSE)
number of inputs	4
number of outputs	2
number of hidden-layer	1
activation function	tansig
training samples	1681
validation samples	360
testing samples	360

**Table 3:** NN performance for different data sets using 25 neurons in the hidden layer.

Data Set	Samples	MSE	R^2^
Training	1681	0.00835	0.99998
Validation	360	0.0207	0.99997
Testing	360	0.0259	0.99997

## Data Availability

Not applicable.

## Abbreviations

The following abbreviations are used in this manuscript:

*a*	wave amplitude
Da	Darcy number
*G*${}_{r}$	nodal value
*H*	step height
Ha	Hartmann number
*k*	thermal conductivity
*L*	length
*n*	unit normal vector
*N*	wave number
Nu	Nusselt number
*p*	pressure
Pr	Prandtl number
Re	Reynolds number
Rew	rotational Reynolds number
*T*	temperature
*u, v, w*	velocity components
*r, z*	cylindrical coordinates
**Greek Characters**
ν	kinematic viscosity
ρ	density of the fluid
ϕ	solid volume fraction
ω	rotational speed
ε	porosity
κ	permeability
Ψ	shape function
**Subscripts**
*c*	cold
*h*	hot
*m*	average
*nf*	nanofluid
*p*	solid particle
**Abbreviations**
ANN	artificial neural net
BFS	backward facing step
CFD	computational fluid dynamics
FP	flow pattern
HT	heat transfer
MaF	magnetic field
MSE	mean squared error
PL	porous layer

## References

[1] Armaly B.F., Durst F., Pereira J., Schönung B. (1983). Experimental and theoretical investigation of backward-facing step flow. J. Fluid Mech..

[2] Chen L., Asai K., Nonomura T., Xi G., Liu T. (2018). A review of Backward-Facing Step (BFS) flow mechanisms, heat transfer and control. Therm. Sci. Eng. Prog..

[3] Li Z., Guo S., Bai H., Gao N. (2019). Combined flow and heat transfer measurements of backward facing step flows under periodic perturbation. Int. J. Heat Mass Transf..

[4] Kherbeet A.S., Safaei M.R., Mohammed H., Salman B., Ahmed H.E., Alawi O.A., Al-Asadi M. (2016). Heat transfer and fluid flow over microscale backward and forward facing step: A review. Int. Commun. Heat Mass Transf..

[5] Xie W., Xi G. (2017). Fluid flow and heat transfer characteristics of separation and reattachment flow over a backward-facing step. Int. J. Refrig..

[6] Hilo A.K., Iborra A.A., Sultan M.T.H., Hamid M.F.A. (2020). Effect of corrugated wall combined with backward-facing step channel on fluid flow and heat transfer. Energy.

[7] Xu J., Zou S., Inaoka K., Xi G. (2017). Effect of Reynolds number on flow and heat transfer in incompressible forced convection over a 3D backward-facing step. Int. J. Refrig..

[8] Saldana J.B., Anand N., Sarin V. (2005). Numerical simulation of mixed convective flow over a three-dimensional horizontal backward facing step. J. Heat Transf..

[9] Williams P., Baker A. (1997). Numerical simulations of laminar flow over a 3D backward-facing step. Int. J. Numer. Methods Fluids.

[10] Lan H., Armaly B., Drallmeier J. (2009). Three-dimensional simulation of turbulent forced convection in a duct with backward-facing step. Int. J. Heat Mass Transf..

[11] Danane F., Boudiaf A., Mahfoud O., Ouyahia S.E., Labsi N., Benkahla Y.K. (2020). Effect of backward facing step shape on 3D mixed convection of Bingham fluid. Int. J. Therm. Sci..

[12] Dol S.S., Salek M.M., Martinuzzi R.J. (2014). Energy redistribution between the mean and pulsating flow field in a separated flow region. J. Fluids Eng..

[13] Selimefendigil F., Öztop H.F. (2017). Forced convection and thermal predictions of pulsating nanofluid flow over a backward facing step with a corrugated bottom wall. Int. J. Heat Mass Transf..

[14] Velazquez A., Arias J., Mendez B. (2008). Laminar heat transfer enhancement downstream of a backward facing step by using a pulsating flow. Int. J. Heat Mass Transf..

[15] Heshmati A., Mohammed H.A., Darus A. (2014). Mixed convection heat transfer of nanofluids over backward facing step having a slotted baffle. Appl. Math. Comput..

[16] Mohammed H., Alawi O.A., Wahid M. (2015). Mixed convective nanofluid flow in a channel having backward-facing step with a baffle. Powder Technol..

[17] Boruah M.P., Pati S., Randive P.R. (2019). Implication of fluid rheology on the hydrothermal and entropy generation characteristics for mixed convective flow in a backward facing step channel with baffle. Int. J. Heat Mass Transf..

[18] Selimefendigil F., Öztop H.F. (2015). Numerical investigation and reduced order model of mixed convection at a backward facing step with a rotating cylinder subjected to nanofluid. Comput. Fluids.

[19] Kumar A., Dhiman A.K. (2012). Effect of a circular cylinder on separated forced convection at a backward-facing step. Int. J. Therm. Sci..

[20] Chatterjee D., Sengupta A., Debnath N., De S. (2015). Influence of an adiabatic square cylinder on hydrodynamic and thermal characteristics in a two-dimensional backward-facing step channel. Heat Transf. Res..

[21] Abu-Nada E. (2008). Application of nanofluids for heat transfer enhancement of separated flows encountered in a backward facing step. Int. J. Heat Fluid Flow.

[22] Mohammed H.A., Fathinia F., Vuthaluru H.B., Liu S. (2019). CFD based investigations on the effects of blockage shapes on transient mixed convective nanofluid flow over a backward facing step. Powder Technol..

[23] Rostami A., Hosseinzadeh K., Ganji D. (2022). Hydrothermal analysis of ethylene glycol nanofluid in a porous enclosure with complex snowflake shaped inner wall. Waves Random Complex Media.

[24] Waqas H., Farooq U., Alqarni M., Muhammad T., Khan M.A. (2021). Bioconvection transport of magnetized micropolar nanofluid by a Riga plate with non-uniform heat sink/source. Waves Random Complex Media.

[25] Hilo A.K., Iborra A.A., Sultan M.T.H., Hamid M.F.A. (2020). Experimental study of nanofluids flow and heat transfer over a backward-facing step channel. Powder Technol..

[26] Manzoor U., Imran M., Muhammad T., Waqas H., Alghamdi M. (2021). Heat transfer improvement in hybrid nanofluid flow over a moving sheet with magnetic dipole. Waves Random Complex Media.

[27] Alsabery A.I., Abosinnee A.S., Ismael M.A., Chamkha A.J., Hashim I. (2021). Natural convection inside nanofluid superposed wavy porous layers using LTNE model. Waves Random Complex Media.

[28] Kakaç S., Pramuanjaroenkij A. (2009). Review of convective heat transfer enhancement with nanofluids. Int. J. Heat Mass Transf..

[29] Kasaeian A., Daneshazarian R., Mahian O., Kolsi L., Chamkha A.J., Wongwises S., Pop I. (2017). Nanofluid flow and heat transfer in porous media: A review of the latest developments. Int. J. Heat Mass Transf..

[30] Ali B., Hussain S., Shafique M., Habib D., Rasool G. (2021). Analyzing the interaction of hybrid base liquid C_2_H_6_O_2_–H_2_O with hybrid nano-material Ag–MoS_2_ for unsteady rotational flow referred to an elongated surface using modified Buongiorno’s model: FEM simulation. Math. Comput. Simul..

[31] Shah T.R., Ali H.M. (2019). Applications of hybrid nanofluids in solar energy, practical limitations and challenges: A critical review. Sol. Energy.

[32] Selimefendigil F., Öztop H.F. (2021). Performance of TEG integrated channel with area expansion by using advanced passive techniques. Int. J. Mech. Sci..

[33] Rasool G., Shafiq A., Durur H. (2021). Darcy-Forchheimer relation in Magnetohydrodynamic Jeffrey nanofluid flow over stretching surface. Discret. Contin. Dyn. Syst..

[34] Selimefendigil F., Öztop H.F. (2022). Impacts of using an elastic fin on the phase change process under magnetic field during hybrid nanoliquid convection through a PCM-packed bed system. Int. J. Mech. Sci..

[35] Salman S., Talib A.A., Saadon S., Sultan M.H. (2020). Hybrid nanofluid flow and heat transfer over backward and forward steps: A review. Powder Technol..

[36] Mohammed H., Al-Aswadi A., Shuaib N., Saidur R. (2011). Convective heat transfer and fluid flow study over a step using nanofluids: A review. Renew. Sustain. Energy Rev..

[37] Atashafrooz M. (2018). Effects of Ag-water nanofluid on hydrodynamics and thermal behaviors of three-dimensional separated step flow. Alex. Eng. J..

[38] Nath R., Krishnan M. (2019). Optimization of double diffusive mixed convection in a BFS channel filled with Alumina nanoparticle using Taguchi method and utility concept. Sci. Rep..

[39] Kherbeet A.S., Mohammed H., Ahmed H.E., Salman B., Alawi O.A., Safaei M.R., Khazaal M. (2016). Mixed convection nanofluid flow over microscale forward-facing step—Effect of inclination and step heights. Int. Commun. Heat Mass Transf..

[40] Nath R., Krishnan M. (2019). Numerical study of double diffusive mixed convection in a backward facing step channel filled with Cu-water nanofluid. Int. J. Mech. Sci..

[41] Selimefendigil F., Öztop H.F. (2020). Effects of local curvature and magnetic field on forced convection in a layered partly porous channel with area expansion. Int. J. Mech. Sci..

[42] Costa V., Raimundo A. (2010). Steady mixed convection in a differentially heated square enclosure with an active rotating circular cylinder. Int. J. Heat Mass Transf..

[43] Khanafer K., Aithal S.M., Vafai K. (2019). Mixed convection heat transfer in a differentially heated cavity with two rotating cylinders. Int. J. Therm. Sci..

[44] Roslan R., Saleh H., Hashim I. (2012). Effect of rotating cylinder on heat transfer in a square enclosure filled with nanofluids. Int. J. Heat Mass Transf..

[45] Abdulrazzaq T., Togun H., Goodarzi M., Kazi S., Ariffin M., Adam N., Hooman K. (2020). Turbulent heat transfer and nanofluid flow in an annular cylinder with sudden reduction. J. Therm. Anal. Calorim..

[46] Alawi O., Sidik N.C., Kazi S., Abdolbaqi M.K. (2016). Comparative study on heat transfer enhancement and nanofluids flow over backward and forward facing steps. J. Adv. Res. Fluid Mech. Therm. Sci..

[47] Geridönmez B.P., Öztop H.F. (2021). Effects of inlet velocity profiles of hybrid nanofluid flow on mixed convection through a backward facing step channel under partial magnetic field. Chem. Phys..

[48] Moayedi H. (2020). Numerical investigation of the effect of oscillating injection nanofluid flow on forced convection heat transfer enhancement over a backward-facing step. Eur. Phys. J. Plus.

[49] Abedalh A.S., Shaalan Z.A., Yassien H.N.S. (2021). Mixed convective of hybrid nanofluids flow in a backward-facing step. Case Stud. Therm. Eng..

[50] Mohammed H., Hussein O.A. (2014). Assisting and opposing combined convective heat transfer and nanofluids flows over a vertical forward facing step. J. Nanotechnol. Eng. Med..

[51] Gautam A.K., Verma A.K., Bhattacharyya K., Mukhopadhyay S., Chamkha A.J. (2021). Impacts of activation energy and binary chemical reaction on MHD flow of Williamson nanofluid in Darcy–Forchheimer porous medium: A case of expanding sheet of variable thickness. Waves Random Complex Media.

[52] Kabeel A., El-Said E.M., Dafea S. (2015). A review of magnetic field effects on flow and heat transfer in liquids: Present status and future potential for studies and applications. Renew. Sustain. Energy Rev..

[53] Abbassi H., Nassrallah S.B. (2007). MHD flow and heat transfer in a backward-facing step. Int. Commun. Heat Mass Transf..

[54] Hussain S., Ahmed S.E. (2019). Unsteady MHD forced convection over a backward facing step including a rotating cylinder utilizing Fe3O4-water ferrofluid. J. Magn. Magn. Mater..

[55] Selimefendigil F., Öztop H.F. (2015). Influence of inclination angle of magnetic field on mixed convection of nanofluid flow over a backward facing step and entropy generation. Adv. Powder Technol..

[56] Sheikholeslami M., Mahian O. (2019). Enhancement of PCM solidification using inorganic nanoparticles and an external magnetic field with application in energy storage systems. J. Clean. Prod..

[57] Kolsi L., Alrashed A.A.A.A., Al-Salem K., Oztop H.F., Borjini M.N. (2017). Control of natural convection via inclined plate of CNT-water nanofluid in an open sided cubical enclosure under magnetic field. Int. J. Heat Mass Transf..

[58] Shafiq A., Rasool G., Alotaibi H., Aljohani H.M., Wakif A., Khan I., Akram S. (2021). Thermally enhanced Darcy-Forchheimer Casson-water/glycerine rotating nanofluid flow with uniform magnetic field. Micromachines.

[59] Sheremet M.A., Oztop H., Pop I., Al-Salem K. (2016). MHD free convection in a wavy open porous tall cavity filled with nanofluids under an effect of corner heater. Int. J. Heat Mass Transf..

[60] Selimefendigil F., Oztop H.F. (2019). Corrugated conductive partition effects on MHD free convection of CNT-water nanofluid in a cavity. Int. J. Heat Mass Transf..

[61] Rasool G., Shafiq A., Hussain S., Zaydan M., Wakif A., Chamkha A.J., Bhutta M.S. (2022). Significance of Rosseland’s radiative process on reactive Maxwell nanofluid flows over an isothermally heated stretching sheet in the presence of Darcy-Forchheimer and Lorentz forces: Towards a new perspective on Buongiorno’s model. Micromachines.

[62] M’hamed B., Sidik N.A.C., Yazid M.N.A.W.M., Mamat R., Najafi G., Kefayati G. (2016). A review on why researchers apply external magnetic field on nanofluids. Int. Commun. Heat Mass Transf..

[63] Sheikholeslami M., Rokni H.B. (2017). Simulation of nanofluid heat transfer in presence of magnetic field: A review. Int. J. Heat Mass Transf..

[64] Atashafrooz M., Sheikholeslami M., Sajjadi H., Delouei A.A. (2019). Interaction effects of an inclined magnetic field and nanofluid on forced convection heat transfer and flow irreversibility in a duct with an abrupt contraction. J. Magn. Magn. Mater..

[65] Selimefendigil F., Öztop H.F. (2020). Hydro-thermal performance of CNT nanofluid in double backward facing step with rotating tube bundle under magnetic field. Int. J. Mech. Sci..

[66] Besanjideh M., Hajabdollahi M., Nassab S.G. (2016). CFD based analysis of laminar forced convection of nanofluid separated flow under the presence of magnetic field. J. Mech..

[67] Aguilar-Madera C.G., Valdés-Parada F.J., Goyeau B., Ochoa-Tapia J.A. (2011). Convective heat transfer in a channel partially filled with a porous medium. Int. J. Therm. Sci..

[68] Poulikakos D., Kazmierczak M. (1987). Forced convection in a duct partially filled with a porous material. J. Heat Transf..

[69] Kuznetsov A. (1999). Fluid mechanics and heat transfer in the interface region between a porous medium and a fluid layer: A boundary layer solution. J. Porous Media.

[70] Vafai K., Kim S. (1990). Fluid mechanics of the interface region between a porous medium and a fluid layer-an exact solution. Int. J. Heat Fluid Flow.

[71] Gibanov N.S., Sheremet M.A., Oztop H.F., Abu-Hamdeh N. (2017). Effect of uniform inclined magnetic field on mixed convection in a lid-driven cavity having a horizontal porous layer saturated with a ferrofluid. Int. J. Heat Mass Transf..

[72] Wang Q., Zeng M., Huang Z., Wang G., Ozoe H. (2007). Numerical investigation of natural convection in an inclined enclosure filled with porous medium under magnetic field. Int. J. Heat Mass Transf..

[73] Revnic C., Grosan T., Pop I., Ingham D. (2011). Magnetic field effect on the unsteady free convection flow in a square cavity filled with a porous medium with a constant heat generation. Int. J. Heat Mass Transf..

[74] Gibanov N.S., Sheremet M.A., Oztop H.F., Al-Salem K. (2017). Effect of uniform inclined magnetic field on natural convection and entropy generation in an open cavity having a horizontal porous layer saturated with a ferrofluid. Numer. Heat Transf. Part A Appl..

[75] Jaiswal S., Yadav P.K. (2019). A micropolar-Newtonian blood flow model through a porous layered artery in the presence of a magnetic field. Phys. Fluids.

[76] Sreenadh S., Prasad K., Vaidya H., Sudhakara E., Krishna G.G., Krishnamurthy M. (2017). MHD Couette flow of a Jeffrey fluid over a deformable porous layer. Int. J. Appl. Comput. Math..

[77] Aly E.H., Ebaid A. (2016). Exact analysis for the effect of heat transfer on MHD and radiation Marangoni boundary layer nanofluid flow past a surface embedded in a porous medium. J. Mol. Liq..

[78] Selimefendigil F., Öztop H.F. (2020). Magnetohydrodynamics forced convection of nanofluid in multi-layered U-shaped vented cavity with a porous region considering wall corrugation effects. Int. Commun. Heat Mass Transf..

[79] Ashorynejad H.R., Zarghami A. (2018). Magnetohydrodynamics flow and heat transfer of Cu-water nanofluid through a partially porous wavy channel. Int. J. Heat Mass Transf..

[80] Shehzad S., Sheikholeslami M., Ambreen T., Shafee A. (2020). Convective MHD flow of hybrid-nanofluid within an elliptic porous enclosure. Phys. Lett. A.

[81] Rashidi M., Abelman S., Mehr N.F. (2013). Entropy generation in steady MHD flow due to a rotating porous disk in a nanofluid. Int. J. Heat Mass Transf..

[82] Kandasamy R., Dharmalingam R., Prabhu K.S. (2018). Thermal and solutal stratification on MHD nanofluid flow over a porous vertical plate. Alex. Eng. J..

[83] Esfe M.H., Arani A.A.A., Rezaie M., Yan W.M., Karimipour A. (2015). Experimental determination of thermal conductivity and dynamic viscosity of Ag-MgO/water hybrid nanofluid. Int. Commun. Heat Mass Transf..

[84] Astanina M.S., Sheremet M.A., Oztop H.F., Abu-Hamdeh N. (2018). MHD natural convection and entropy generation of ferrofluid in an open trapezoidal cavity partially filled with a porous medium. Int. J. Mech. Sci..

[85] Yousofvand R., Derakhshan S., Ghasemi K., Siavashi M. (2017). MHD transverse mixed convection and entropy generation study of electromagnetic pump including a nanofluid using 3D LBM simulation. Int. J. Mech. Sci..

[86] Sankar M., Venkatachalappa M., Shivakumara I. (2006). Effect of magnetic field on natural convection in a vertical cylindrical annulus. Int. J. Eng. Sci..

[87] Lewis R.W., Nithiarasu P., Seetharamu K.N. (2004). Fundamentals of the Finite Element Method for Heat and Fluid Flow.

[88] Heinrich J.C., Pepper D.W. (2017). Intermediate Finite Element Method: Fluid Flow and Heat Transfer Applications.

[89] Reddy J.N., Gartling D.K. (2010). The Finite Element Method in Heat Transfer and Fluid Dynamics.

[90] Khanafer K., Al-Azmi B., Al-Shammari A., Pop I. (2008). Mixed convection analysis of laminar pulsating flow and heat transfer over a backward-facing step. Int. J. Heat Mass Transf..

[91] Carrington D.B., Pepper D.W. (2002). Convective heat transfer downstream of a 3-D backward-facing step. Numer. Heat Transf. Part A Appl..

[92] Selimefendigil F., Öztop H.F. (2018). Laminar convective nanofluid flow over a backward-facing step with an elastic bottom wall. J. Therm. Sci. Eng. Appl..

[93] Zhang T., Che D. (2016). Double MRT thermal lattice Boltzmann simulation for MHD natural convection of nanofluids in an inclined cavity with four square heat sources. Int. J. Heat Mass Transf..

[94] Baytaş A. (2000). Entropy generation for natural convection in an inclined porous cavity. Int. J. Heat Mass Transf..

[95] Saeid N.H., Pop I. (2004). Transient free convection in a square cavity filled with a porous medium. Int. J. Heat Mass Transf..

[96] Khandelwal V., Dhiman A., Baranyi L. (2015). Laminar flow of non-Newtonian shear-thinning fluids in a T-channel. Comput. Fluids.

[97] Aggarwal C.C. (2018). Neural networks and deep learning. Springer.

[98] Nunes I., Da Silva H.S. (2018). Artificial Neural Networks: A Practical Course.

[99] Jain L.C. (1999). Recurrent Neural Networks: Design and Applications.

[100] Hagan M.T., Menhaj M.B. (1994). Training feedforward networks with the Marquardt algorithm. IEEE Trans. Neural Netw..

[101] Kalogirou S. (1999). Applications of artificial neural networks in energy systems A review. Energy Convers. Manag..

